# Retrospective Study of Intestinal Parasites Among Pediatric Patients Treated at a Tertiary Hospital in Southern Ethiopia: A 5-Year Retrospective Study

**DOI:** 10.1155/jotm/6688437

**Published:** 2025-11-22

**Authors:** Temesgen Anjulo Ageru, Feseha Ayele, Kaleb Dana Hebana, Yisehak Kussa, Muhammad Haroon Stanikzai, Francis Walugembe, Aman Buche Shano

**Affiliations:** ^1^College of Medicine and Health Sciences, Wolaita Sodo University, P.O. Box 138, Wolaita Sodo, South Ethiopia, Ethiopia; ^2^Department of Public Health, Kandahar University, Kandahar, Afghanistan; ^3^Department of Environmental Health, Kampala International University, Kampala, Uganda; ^4^Department of Biotechnology, Wolaita Sodo University, Wolaita Sodo, South Ethiopia, Ethiopia

**Keywords:** Ethiopia, helminths, parasite, pediatrics, protozoa, Wolaita

## Abstract

**Background:**

In Ethiopia, parasitic infections pose a major health concern, particularly affecting children. Understanding the occurrence of parasitic infections within a hospital setting provides insights into the overall health status of the broader community.

**Aim:**

The aim of this study was to evaluate the occurrence of parasitic infections among pediatric patients treated at a tertiary hospital in South Ethiopia over a 5-year period.

**Method:**

A retrospective data collection was conducted from February 1, 2023, to April 1, 2023, using the parasitology laboratory stool test results logbook at Wolaita Sodo University Comprehensive Specialized Hospital, a tertiary hospital. The data covered the period from January 1, 2018, to December 30, 2022. Microsoft Excel was used to enter data from logbooks, and the descriptive results were summarized using tables and graphs.

**Result:**

Out of 6542 pediatric patients examined, 6482 were included in the evaluation after excluding incomplete records. Among these patients, 51.6% tested positive for at least one parasite. Of the positive cases, 68% were due to protozoan infections and 32% were due to helminth infections. The most identified parasite was *Entamoeba histolytica/dispar*, accounting for 43.7% of positive cases. *Ascaris lumbricoides* was the predominant helminth, representing 17.2% of positive cases. The age group under 5 years had the highest parasitic infections among others.

**Conclusion:**

The occurrence of parasitic infections among pediatric patients treated at this hospital was alarmingly high. Therefore, it is recommended to promote personal hygiene, enhance environmental sanitation, and implement regular screening for intestinal parasites.

## 1. Introduction

Parasitic infections are a significant public health concern worldwide, particularly in developing countries where high occurrence rates have been documented across various regions [1]. According to the World Health Organization (WHO), an estimated 3.5 billion individuals globally are afflicted by intestinal parasites, with up to 178 million children experiencing undernourishment and 3.5–5 million deaths occurring annually [2–4]. These infections are particularly widespread in low-income countries, primarily in tropical and subtropical areas, where factors such as fecal contamination of water and food, hot and humid climates, and environmental and hygiene-related issues enhance the transmission of these parasites [3, 5].

*Ascaris lumbricoides*, hookworms, and *Trichuris trichiura* infect approximately one-fourth of the world's population, affecting 250 million, 151 million, and 45 million people, respectively [6, 7]. These parasites are responsible for a significant number of deaths, causing approximately 65,000, 60,000 and 70,000 fatalities, respectively [8].

Protozoan infections also cause critical public health issues, often leading to iron deficiency anemia, growth stunting, and various physical and mental health problems among children [9]. These infections result in nutritional depletion, reduced immunity in infants, mucosal damage, lymphatic leakage, and local hemorrhage [3, 10].

The impact of parasitic infections on the host's immune system can occur through direct competition for niche space within the gastrointestinal tract and host immune modulation. Direct immune responses triggered by parasites primarily involve the downregulation of inflammation, favoring a protective response [11]. These changes support resistance and tolerance mechanisms of the parasite by creating conditions that allow for its survival and extended reproductive phase. The excretory products that are remitted by live worms can also serve as an indirect means of parasitic contact with host immune cells [12, 13].

Up to 95% of individuals in Sub-Saharan African countries have an intestinal parasitic infection, which is alarmingly significant. Approximately 250 million individuals in this region are believed to be infected with at least one parasite [14, 15]. This high frequency of occurrence is observed predominantly in individuals with limited financial resources, residing in developing regions characterized by poor living conditions, inadequate environmental sanitation, and contaminated water supplies [16, 17].

Schistosomiasis, known as bilharzia, is a parasitic disease caused by Schistosoma worms. It is prevalent in Sub-Saharan Africa, where millions of individuals are affected. In this region, schistosomiasis alone leads to more than 534,000 deaths and an estimated 57 million disability, while *Entamoeba histolytica/dispar* contributes to approximately 100,000 deaths each year [18, 19].

Similar to other developing countries, intestinal parasites are a major cause of public health issues in Ethiopia and account for a substantial portion of outpatient morbidity [15, 20]. In the country, about 81 million live in endemic areas, with approximately 52%–65% of children infected with parasites [21, 22]. Specifically, 9.1 million preschool children and more than 25 million school children are exposed to these parasites [23]. Moreover, 33.9% of children in Ethiopia are anemic due to intestinal parasitic infections [9]. These infections contribute to 46.3% of childhood undernutrition [24].

Despite some investigations aimed at assessing the occurrence of intestinal parasites in various regions of the country, comprehensive data on the pediatric population at the tertiary hospital remain unavailable, including the present study setting. Therefore, this study aims to evaluate the occurrence of parasitic infection among pediatric patients treated at Wolaita Sodo University Comprehensive Specialized Hospital.

The findings from this study are expected to enhance screening practices, prevention, and control strategies in the study area and similar settings. The outcomes will provide valuable insights for designing and implementing cost-effective interventions, ultimately enabling healthcare providers to deliver optimal care to pediatric patients. Furthermore, policymakers can utilize this study's evidence to guide actions in the management of parasitic infections. Last, this study serves as a resource for researchers interested in investigating the occurrence of this disease.

## 2. Materials and Methods

### 2.1. Study Area

**Figure 1:**
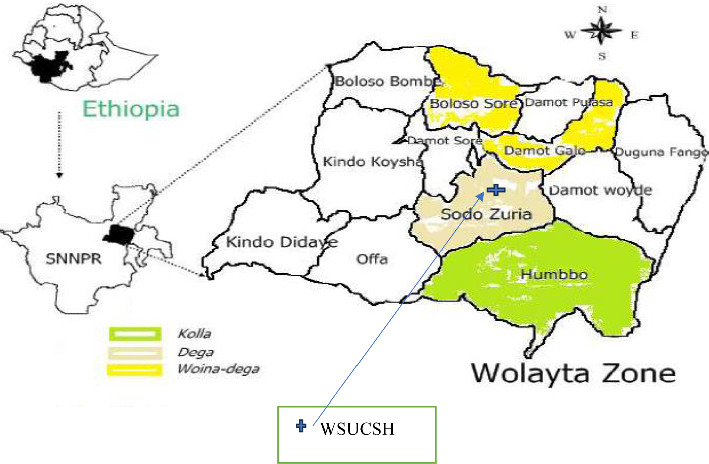
Map of Sodo Town and Wolaita Sodo University Comprehensive Specialized Hospital (WSUCSH).

This study was carried out at Wolaita Sodo University Comprehensive Specialized Hospital, situated in Sodo Town, Wolaita Zone, Southern Ethiopia. It is situated 329 km away from Addis Ababa, the capital of Ethiopia, and 3 km from Wolaita Sodo, the capital city of the Southern Region of Ethiopia (see study area map, Figure 1). The hospital was established in 1928, and currently, over 15 million individuals are served by the hospital. With more than 370 beds, the hospital's departments such as gynecology and obstructive, medical, surgical ward, oncology, dialysis, dental unit, ophthalmology, laboratory, pharmacy, radiology, and pediatrics are within its important wards.

### 2.2. Study Design and Period

A retrospective cross-sectional study covering the previous 5 years from January 1, 2018, to December 30, 2022, was carried out from February 1 to April 1, 2023.

### 2.3. Source and Study Population

The source population consisted of all pediatric patients who visited Wolaita Sodo University Comprehensive Specialized Hospital between January 1, 2018, and December 30, 2022. This study population comprised pediatric patients who gave stool specimens and were examined for intestinal parasites and had complete records of age, sex, year of attendance, and stool examination results during the study duration.

### 2.4. Exclusion and Inclusion Criteria

Pediatric patients included were all pediatric patients examined for stool samples with complete data on age, sex, and stool examination result from January 1, 2018, to December 30, 2022. Those with incomplete data were excluded from the study.

### 2.5. Study Variables

The dependent variable was the occurrence of intestinal parasites, while the independent variables included age, sex, and year of attendance.

### 2.6. Operational Definition

#### 2.6.1. Parasite

A parasite that resides in the human intestinal tract and bloodstream.

#### 2.6.2. Pediatric Patient

An individual below 15 years, who received stool examination services at Wolaita Sodo University Comprehensive Specialized Hospital.

### 2.7. Parasitological Diagnostic Methods of Wet Mount

A diagnostic method was conducted according to the hospital's Internal Standard Operating Procedure (unpublished). The hospital used saline (0.85%) as a wet mount diagnostic method for identifying intestinal parasites. When a physician orders stool samples for an examination, pediatric patients come to the laboratory with a stool request format. Then, laboratory technologists inform them about the stool examination procedure and order patients to bring stool.

#### 2.7.1. Stool Specimen Collection and Storage

1. Patients are instructed to collect in a dry, clean, and leak-proof container.
2. A laboratory technologist ensures no urine, water, soil, or other materials contaminate the container.
3. Specimens should be collected before administrations of antibiotic, kaolin, mineral oil, nonabsorbable antidiarrheal preparations, barium or bismuth (clearance needed 7–10 days), and antimicrobial agents (2–3 weeks), as these substances render the specimen unsatisfactory for examinations.
4. Fresh stool samples should be examined, processed, or preserved immediately.

#### 2.7.2. Quality Assurance in the Wet Mount Method

A hospital performs daily quality control of normal saline (0.85%). A drop of normal saline is placed on a clean microscopic slide, checked for the presence or absence of any unusual material, and documented daily. Positive samples must be preserved and stored for checking the performance of the microscopic objectives.

#### 2.7.3. Macroscopic and Microscopic Procedures

1. Consistency of the stool specimen such as formed, soft, loose, or watery was assessed. Those with blood and mucus were examined first, followed by liquid specimens, if several specimens are received together.
2. Material such as a microscopic slide was labeled.
3. The specimen was placed on a microscopic slide.
4. A solid stool specimen was prepared by using a drop of saline and iodine to get better results.
5. The prepared smear was covered with a slip to prevent the preparation from drying and the organism from moving.
6. The entire smear area was examined, with a 10x objective; if something suspicious was seen, a higher magnification (40x) was used.

### 2.8. Data Collection and Quality Assurance

Demographic data of pediatric patients, stool examination results, and year of attendance in the hospital were extracted by oriented laboratory technologists using an extraction tool. A data extraction tool was developed in English based on similar studies. All recorded information was cross-checked for accuracy. Data collectors were trained in data extraction methods, closely supervised during data extraction from the logbook, and checked for completeness and consistency. Incomplete data were excluded.

### 2.9. Data Analysis

The data collected from the laboratory logbook was entered into Microsoft Excel 2019. Tables and graphs were used to present the frequency of parasites, along with descriptive statistics.

### 2.10. Ethical Consideration and Participants' Consent

The ethical clearance letter was obtained from the Ethical Review Committee of Wolaita Sodo University College and Health Sciences. The committee approved the proposal from an ethics point and recommended collecting retrospective data from Wolaita Sodo University Comprehensive Specialized Hospital. Furthermore, the committee also waived the consent form, as secondary data were analyzed in this study. All procedures in this study adhere to the Declaration of Helsinki principles.

## 3. Results

A total of 6542 pediatric patients were treated at Wolaita Sodo University Comprehensive Specialized Hospital for stool examination results from January 1, 2018, to December 30, 2022. Among these, 60 pediatric patients had incomplete records and were excluded from the study. The remaining 6482 patients, with complete demographic information, were evaluated from the parasitological results registration logbook. In terms of gender distribution, males comprised 68.1% of the total pediatric patients, while females constituted 31.9% in the 5-year period.

Out of the total patients, 3346 (51.6%) tested positive for at least one intestinal parasite. The prevalence of intestinal parasites varied across the years of pediatric patients' visits, with the highest rates of 69.6% and 61.3% in 2022 and 2020, respectively. The identified intestinal parasites include *Entamoeba histolytica/dispar, Giardia intestinalis,* hookworm species*, Ascaris lumbricoides, Schistosoma mansoni, Taenia species, Hymenolepis nana,* and *Strongyloides stercoralis.* The two most prevalent parasites were *Entamoeba histolytica/dispar, found in 43.*7% of positive cases, 22.4% of all patients examined, and *G. intestinalis*, found in 25.2% of positive patients, 13% of all patients examined. The most prevalent helminth was *A. lumbricoides,* accounting for 17.2% of the positive cases and 8.9% of all examined. The least identified parasites were *S. stercoralis and S. mansoni* (Table 1).

### 3.1. Classification of Intestinal Parasites

**Figure 2:**
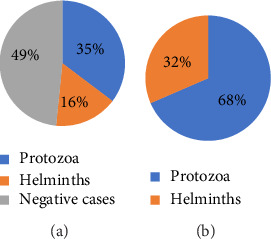
(a) Proportions of parasites among pediatric patients treated and (b) proportion of parasites within positive cases at Wolaita Sodo University Comprehensive Specialized Hospital (2018–2022).

Classification of identified intestinal parasites revealed two main categories: protozoa and helminths. Among pediatric patients tested, the protozoa identified included *E. histolytica/dispar* and *G. intestinalis*. These two parasites accounted for 35.3% (2291/6482) of all cases tested, constituting 68.5% (2291/3346) of positive cases. The identified helminths were *A. lumbricoid*es, *H. nana, tapeworm species,* hookworms*, S. stercoralis*, and *S. mansoni*, collectively accounting for 16.3% (1055/6482) of total patients examined and 31.5% (1055/3346) of positive cases (Figures 2(a) and 2(b)).  Figure 2(a) illustrates the proportion of intestinal parasites among pediatric patients treated, while Figure 1(b) demonstrates the proportion of parasites within positive cases at Wolaita Sodo University Comprehensive Specialized Hospital from 2018 to 2022 (Figures 2(a) and 2(b)).

### 3.2. The Trend Frequency of Intestinal Parasites by Year

The occurrence of intestinal parasites was observed over a 5-year period among pediatric patients. During this time, a total of 6482 pediatric patients were examined for intestinal parasites; of these, 3346 patients tested positive for intestinal parasites or at least one parasitic infection. About 37.7% of positive cases were detected in the year 2018, whereas 69.6% were detected in the year 2022.

**Figure 3:**
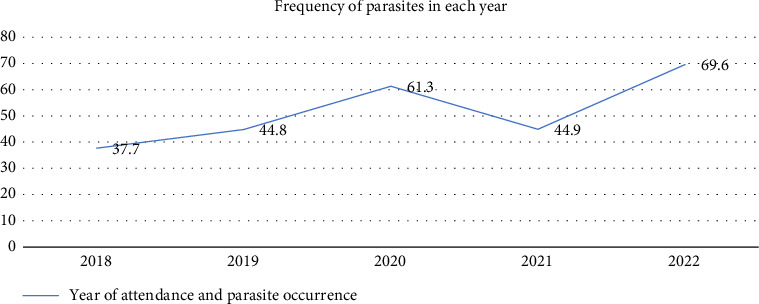
Frequency of parasites in each year among pediatric patients treated at Wolaita Sodo University Comprehensive Specialized Hospital (2018–2022).

Figure 2 illustrates the frequency of intestinal parasites within each year among pediatric patients treated at Wolaita Sodo University Comprehensive Specialized Hospital from 2018 to 2022 (Figure 3).

### 3.3. Age Groups and Prevalence of Intestinal Parasites

The frequency of intestinal parasites was observed across different age groups. Among the 3346 (51.6%) identified positive pediatric patients, approximately 31.70% in pediatrics were under 5 years old, 17% in the 5–8 years range, 22.23% in the 9–12 years range, and 28.96% in those aged above 12 years. The age group most affected by all parasites was children under 5 years old. The most identified intestinal parasite across all age groups was *E. histolytica/dispar*, accounting for 43.7% of all positive cases (Table 2).

### 3.4. The Occurrence of Intestinal Parasites by Gender

Among the 3346 pediatric patients who tested positive for intestinal parasites, 2570 (76.8%) were male and 776 (23.2%) were female. The most common parasites identified among males were E. *histolytica/dispar*, 34.2%, and *Giardia intestinalis*, 18.5%, while among females, these parasites were found at lower frequencies of 9.2% and 6.6%, respectively. Out of the 4417 males tested, 2570 were positive, resulting in a positivity rate of 58.2%. Among the 2065 females tested, 776 were positive, yielding a positivity rate of 37.60% (Table 3).

## 4. Discussion

Parasitic infections present a significant health challenge in low-income countries such as Ethiopia [25]. Factors such as poor personal hygiene, overcrowding, limited access to clean water, inadequate health education, and poverty contribute to the occurrence of these infections [26].

This retrospective study conducted at Wolaita Sodo University Comprehensive Specialized Hospital aimed to evaluate the occurrence of intestinal and bloodstream parasites among pediatric patients treated between January 1, 2018, and December 30, 2022. A total of 6482 pediatric patients, with complete demographic information, were included; of whom, 51.6% tested positive for at least one parasite.

The prevalence observed in this study is notably higher than that reported in previous Ethiopian studies conducted in northwest, 42.2% [14], southeast, 38.5% [26], and southern regions, 39.9% [27]. These variations could be attributed to differences in study population such as hospital-based symptomatic children vs. community samples, sample sizes, seasonal timing, and diagnostic methods. In addition, our study was based in a tertiary care hospital, where symptomatic and clinically ill children are more likely to present, potentially inflating the observed prevalence due to sampling bias.

Our findings are consistent with studies conducted in India, 50.8% [28], and systematic reviews from Ethiopia, 48.2% [29] and 53.3% [30], further suggesting a high burden of intestinal parasitic infections in pediatric hospital settings. However, our observed prevalence is lower than figures reported in other LMICs, such as South Africa, 64.8% [31]; Ethiopia, 65.4% [22]; Burkina Faso, 84.7% [32]; Nigeria, 80.9% [33]; and Sao Tome, 64.7% [34]. These higher rates may be due to more intense transmission in certain ecological zones or the use of more sensitive diagnostic methods such as concentration techniques or multiple-sample protocols. In contrast, our study relied solely on wet mount microscopy with a single stool sample, which may underestimate true occurrence, especially for protozoan infections, where parasite load can be low.

The higher occurrence of *Entamoeba histolytica/dispar* and *Giardia intestinalis* found in this study aligns with similar findings from hospital-based studies in Somalia [35], India [28], Ethiopia [36], and Egypt [37]. This similarity reinforces the role of poor environmental and personal sanitation, particularly in urban slums or peri-urban areas, where fecal–oral transmission pathways remain prevalent.

Intestinal parasites affected all age groups in our study; however, children under 5 years old were the most affected (31.70%), followed by those over 12 years old (28.96%). This is an agreement with other studies from Somalia [35], northern Ethiopia [38], and other LMICs [39], where younger children are particularly vulnerable due to their immature immune system and higher exposure to unhygienic environments such as playing in contaminated soil and poor hand hygiene. Children in this age group also tend to have closer contact with caregivers and contaminated household environments, further increasing their risk of infection.

Our study also found a higher infection rate among male children. This contrasts with a study from a tertiary hospital in Haryana, India [1], which found higher rates among females. However, the gender imbalance in our sample (68.1% male) may have contributed to this observation. Still, the gender disparity is an important consideration for future research and program design, as behavioral and cultural factors such as play habits and outdoor exposure may differ between male and female children and influence infection risks.

Furthermore, our five-year trend showed an increasing pattern in some years. The proportion of intestinal parasitic infections among pediatric patients was 37.70% in 2018, 44.80% in 2019, 61.30% in 2020, 44.90% in 2021, and reached a peak of 69.6% in 2022. The rising trend, particularly the high prevalence in 2020 and 2022, may suggest environmental influence. Furthermore, the dominance of *E. histolytica/dispar* and *G. intestinalis* signifies the need to prioritize these infections in public health interventions.

Lastly, unlike some studies that reported polyparasitism, studies conducted in Dessie hospital reported dual infections in 1.2% of children [38]; however, our dataset did not capture whether individuals were infected with more than one parasite. Including such data for future research would provide a more nuanced picture of parasitic burden and clinical impact.

In summary, this study's significance in existing literature lies in its provision of localized, context-specific, and updated data. Such information can facilitate a better understanding and management of intestinal parasitic infections among pediatric patients in the study region. This might guide future research and public health efforts within the field of parasitology and pediatric healthcare.

## 5. Strengths and Limitations

### 5.1. Strengths of the Study

1. Large sample size: The study analyzed a substantial number of pediatric patients (6482) over a 5-year period, providing a comprehensive overview of intestinal parasite occurrence in this population.
2. Demographic diversity: The study included patients with complete demographic information, allowing for an analysis of gender-specific frequency rates and age-related susceptibility, providing valuable insights into demographic variations.
3. Detailed parasitological analysis: The study conducted a thorough parasitological examination, identifying various types of intestinal parasites, including protozoa and helminths, providing a comprehensive understanding of the prevalent parasites.
4. Identification of high-risk groups: By categorizing age groups and genders, the study identified vulnerable populations, such as those under 5 years old and males, prone to higher infection rates, thus guiding targeted intervention strategies.
5. Public and clinical relevance: The findings hold clinical significance by highlighting specific parasites such as *E. histolytica/dispar* and *G. intestinalis* with higher prevalence rates, aiding in prioritizing treatment and preventive measures.

### 5.2. Limitations of the Study

1. Single-center study: The study was conducted in a tertiary hospital, where patients come from various parts of the country to be seen. However, this limits the generalizability of the findings to a broader population. Parasitic occurrences may vary in different geographical regions or healthcare settings.
2. Diagnostic sensitivity: The study relied on a single stool specimen and primarily 10x and 40x magnification, which may limit sensitivity, especially for protozoan parasites. No confirmatory tests were performed to differentiate *E. histolytica* from *E. dispar*, limiting pathogenic interpretation.
3. Environmental and behavioral factors: The study did not extensively explore environmental or behavioral factors (such as sanitation, water source, and dietary habits) that could significantly influence parasite transmission and occurrence.
4. Incomplete parasite spectrum: The study identified several common parasites; due to the retrospective nature of the study, it did not mention dual or triple parasitic infection, which could provide valuable insights into the effectiveness of current practices.
5. Exclusion of incomplete records: Excluding 60 pediatric patients with incomplete records might affect the representation of the data, potentially influencing the observed prevalence rates.

Acknowledging the limitation by incorporating multicenter studies, using more sensitive diagnostic tests such as the concentration technique, incorporating sanitation, water source, dietary habits, and a wider spectrum of parasites can enhance the depth and applicability of future research in this field.

## 6. Conclusion and Recommendation

This study, conducted at Wolaita Sodo University Comprehensive Specialized Hospital, presents significant insights into the occurrence of intestinal parasites among pediatric patients in Ethiopia, highlighting an infection rate of 51.6%. The high occurrence of the infection of *E. histolytica/dispar and G. intestinalis* suggest that fecal–oral transmission linked to poor sanitation remains a critical public health issue. The disproportionally high infection rates among children under 5 years old emphasize specific vulnerability groups within populations.

Healthcare providers conduct regular screenings and diagnostic tests for intestinal parasites among vulnerable groups and provide targeted treatment and education on preventive measures to affected patients and their families.

The government and policymakers should allocate resources toward improving sanitation infrastructure, access to clean water, and healthcare services in underserved regions.

Public health officials should establish surveillance systems to monitor and track the prevalence of intestinal parasites regularly and create targeted intervention programs that address the specific needs of high-risk groups, considering age, gender, and geographical location.

Researchers should conduct further studies to explore the dynamics of intestinal parasitic infections, considering environmental factors, seasonal variations, and emerging trends.

## Figures and Tables

**Table 1:** The frequency of intestinal parasites stratified by year among pediatric patients treated at Wolaita Sodo University Comprehensive Specialized Hospital (2018–2022).

No	Types of parasite	Year of attendance					Total
		2018	2019	2020	2021	2022	
		*N* (%)	*N* (%)	*N* (%)	*N* (%)	*N* (%)	*N* (%)
1	*E. histolytica/dispar*	249 (20.60)	302 (26.80)	313 (22.0)	290 (17.80)	298 (27.20)	1452 (43.70)
2	*G. intestinalis*	102 (8.40)	88 (7.80)	281 (19.8)	127 (7.80)	241 (21.90)	839 (25.20)
3	*A. lumbricoides*	36 (3.0)	47 (4.20)	111 (7.80)	238 (14.6)	143 (13.0)	575 (17.20)
4	*H. nana*	17 (1.41)	12 (1.10)	39 (2.80)	21 (1.3)	9 (0.82)	207 (6.20)
5	*Taenia* spp.	13 (1.10)	19 (1.70)	27 (1.90)	14 (0.9)	31 (2.80)	104 (3.10)
6	Hookworms	32 (2.70)	17 (1.50)	81 (6.40)	36 (2.2)	41 (3.70)	98 (2.90)
7	Others	7 (0.60)	20 (1.80)	18 (1.30)	5 (0.3)	1 (0.10)	51 (1.50)
	Total parasites identified	456 (37.7)	505 (44.80)	870 (61.30)	731 (44.9)	764 (69.6)	3346 (100%)
	Patient examined	1209	1128	1420	1628	1097	3346/6482 (51.6)

*Note:* Others include *S. stercoralis* and *S. mansoni*.

**Table 2:** Age groups and types of intestinal parasites among pediatric patients treated at Wolaita Sodo University Comprehensive Specialized Hospital (2018–2022).

Types of parasite	Age group of children and infection rates				Total
	< 5 year (%)	5–8 years (%)	9–12 years (%)	> 12 years (%)	
*E. histolytica/dispar*	9.10	5.60	13.0	16.0	43.70%
*G. intestinalis*	11.40	5.40	5.0	3.40	25.20%
*A. lumbricoides*	6.20	3.40	1.60	6.0	17.20%
Hookworms	1.70	1.40	1.90	1.20	6.20%
*Taenia species*	1.40	0.50	0.40	0.80	3.10%
*H. nana*	1.30	0.30	0.10	1.20	2.90%
Others	0.54	0.40	0.20	0.40	1.50%
Total	31.7	17.0	22.20	29.0	100

*Note:* Others include *S. stercoralis* and *S. mansoni*.

**Table 3:** The frequency of occurrence of parasites by gender among pediatrics patients at Wolaita Sodo University Comprehensive Specialized Hospital (2018–2022).

Parasites identified	Male		Female		Total	
	(*N*)	(%)	(*N*)	(%)	(*N*)	(%)
*E. histolytica/dispar*	1144	34.20	308	9.20	1452	43.70
*G. intestinalis*	618	18.50	221	6.60	839	25.20
*A. lumbricoides*	478	14.30	97	2.9	575	17.20
Hookworms	191	5.70	32	0.95	207	6.60
*Taenia species*	67	2.0	41	1.20	104	3.20
*H. nana*	31	0.90	67	2.0	98	2.90
Others	41	1.20	10	0.30	51	1.50
Total	2570	76.80	776	23.20	3346	100

*Note: N* = number; % = percentage. Others include *S. stercoralis* and *S. mansoni*.

## Data Availability

All necessary data for this research are included in the manuscript.

## Nomenclature

IPIs: Intestinal parasitic infections
WHO: World Health Organization
WSUCSH: Wolaita Sodo University Comprehensive Specialized Hospital
GIT: Gastrointestinal tract
IAC: Internationally adopted children
STHs: Soil-transmitted helminths
